# A Novel Adaptive Level Set Segmentation Method

**DOI:** 10.1155/2014/914028

**Published:** 2014-09-01

**Authors:** Yazhong Lin, Qian Zheng, Jiaqiang Chen, Qian Cai, Qianjin Feng

**Affiliations:** ^1^The 175 Hospital, Southeast Hospital of Xiamen University, Zhangzhou, Fujian 363000, China; ^2^Zhengzhou University of Light Industry, Zhengzhou, Henan 450002, China; ^3^Southern Medical University, Guangzhou, Guangdong 510515, China

## Abstract

The adaptive distance preserving level set (ADPLS) method is fast and not dependent on the initial contour for the segmentation of images with intensity inhomogeneity, but it often leads to segmentation with compromised accuracy. And the local binary fitting model (LBF) method can achieve segmentation with higher accuracy but with low speed and sensitivity to initial contour placements. In this paper, a novel and adaptive fusing level set method has been presented to combine the desirable properties of these two methods, respectively. In the proposed method, the weights of the ADPLS and LBF are automatically adjusted according to the spatial information of the image. Experimental results show that the comprehensive performance indicators, such as accuracy, speed, and stability, can be significantly improved by using this improved method.

## 1. Introduction

Since the introduction by Kass et al. [1], active contour models (ACMs) have been widely used in image segmentation [2–4]. The existing ACMs based on the level set method initially proposed to handle the topological changes during the curve evolution can be broadly classified as either edge-based models [5–7] or region-based models [8–15] according to the type of adopted image features. The basic idea of the level set method is to implicitly embed the moving contour into a higher dimensional level set function and view the contour as its zero level set [16]. ACMs have desirable properties over the conventional image segmentation methods, such as thresholding, edge detection, and region growing. First, ACMs can provide closed and smooth contours in segmentation results, which are necessary for further application such as shape analysis and recognition. Second, ACMs can get the object boundaries with subpixel accuracy.

**Figure 1:**
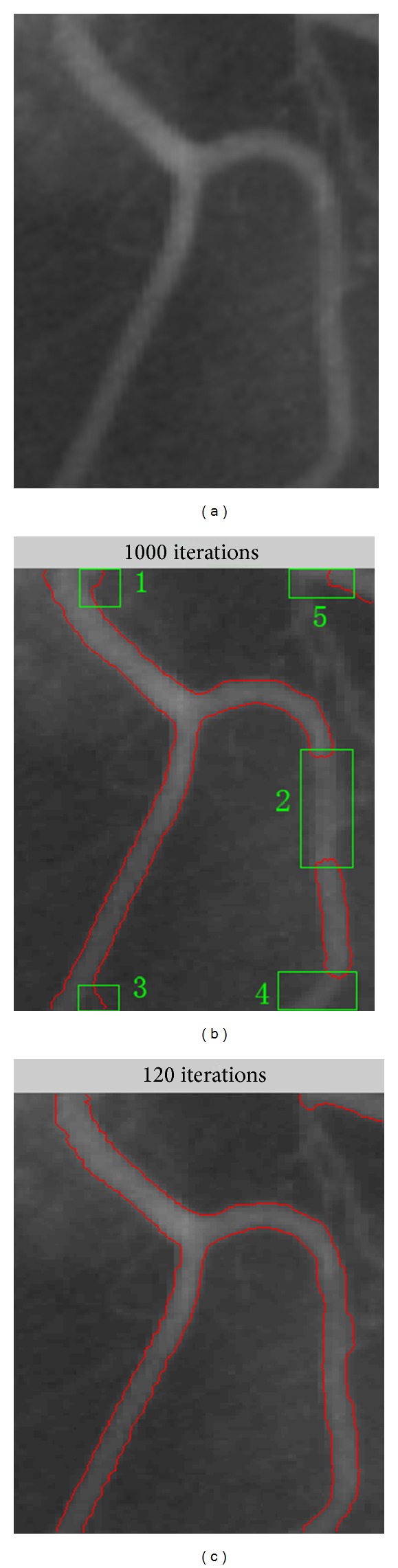
The vessel segmentation of an X-ray image with the two methods. (a) The original image. (b) The result of the ADPLS method. (c) The result of the LBF method.

Edge-based models utilize image gradient to stop evolving contours on the object boundaries. Recently, He et al. proposed adaptive distance preserving level set (ADPLS) [7] evolution for image segmentation, in which the initial curve is no longer required to surround (let in or keep out) the objects to be detected. Moreover, starting with only one initial curve whose position is anywhere in the image, it can automatically detect interior and exterior contours of an object and edges of multiobjects. In addition, a large time step can be used to speed up the curve evolution in the numerical solution of the partial differential equation. However, this method does not contain any local intensity information, which is crucial for segmentation of images with intensity inhomogeneity. As a consequence, the ADPLS method generally fails to segment images with significant intensity inhomogeneity, which is illustrated in the following vessel segmentation in Figure 1.  Figure 1(a) is a typical image with intensity inhomogeneity, and Figure 1(b) shows the final segmentation result of the ADPLS method. It is obvious that boundary leakage arises in regions 1 and 3, and less segmentation arises in regions 2, 4, and 5. This example shows the inability of the ADPLS method to segment images with intensity inhomogeneity.

To guide the motion of the active contour, the region-based models identify each region of interest by using a certain region descriptor such as intensity, color, texture, or motion. Region-based models have better performance than edge-based models in the presence of weak boundaries. Local binary fitting model (LBF) [14] is one of the classical region-based models. It can not only segment the image with weak boundaries well, but also overcome the segmentation error caused by intensity inhomogeneity. The LBF model draws upon spatially varying local region information; thus it is able to deal with intensity inhomogeneity.  Figure 1(c) shows the final segmentation result of the LBF model, which shows much better results than that of the ADPLS method. However, compared to the ADPLS method, the LBF model also has its own disadvantages. First, the LBF model is sensitive to the initial contour, and inappropriate initial contours might lead to failure of segmentation. Second, its curve evolution is slow due to some limitation of the time step.

In this paper we proposed a novel adaptive level set method to combine the good properties of both ADPLS and LBF methods. In a simultaneous and automatic way, the proposed method adjusts the proportion of the ADPLS and LBF methods according to spatial image information adaptively. As a consequence, the advantages of the ADPLS method and the LBF method are exploited with minimized disadvantages. The following experiments on both simulated and real images show that the proposed method can achieve segmentation with higher accuracy.

## 2. Backgrounds

### 2.1. The ADPLS Method

Let *Ω* ⊂ *R* ^2^ be the image domain, and let *I* : *Ω* → *R* be a given gray level image. The ADPLS method [7] was recently proposed to overcome the disadvantage of the distance preserving level set method proposed by He et al., which requires the initial curve surrounding (let in or keep out) the objects to be detected. It imports a variable weighting coefficient whose sign symbol and size are adjusted by image information. So the zero level set can choose its evolution direction adaptively.

In image segmentation, active contours are dynamic curves that move toward the object boundaries by minimizing a predefined energy functional. Let *g* be the edge indicator function defined by

(1) $$\begin{array}{c} g\left(\nabla I\right)=\exp\left(-\frac{\left|\nabla G_{\sigma }\times I\right|}{m}\right), \end{array}$$

where *G*_*σ*_ is the Gaussian kernel with standard deviation *σ* and *m* > 0 is a constant.

In the ADPLS method, a variational framework on the level set function *ϕ* is defined as follows:

(2) εADPLS(ϕ)=α12∫Ω(|∇ϕ|−1)2dx dy+β∫Ωgδ(ϕ)|∇ϕ|dx dy+v(I)∫ΩgH(−ϕ)dx dy,

where the first term is the internal energy of *ϕ* that characterizes the deviation of the level set function from a signed distance function, *α* > 0 is the weight of this internal energy term, the second term computes the length of the zero level curve of *ϕ*, *g* is the edge indicator function defined by (1), *δ* is the univariate Dirac function, *H* is the Heaviside function, and *β* > 0 is the weight of the second term. The definition of *v*(*I*) is given by

(3) $$\begin{array}{c} v\left(I\right)=c\cdot \mathrm{sgn}\left(\Delta G_{\sigma }\times I\right)\cdot \left|\nabla G_{\sigma }\times I\right|, \end{array}$$

where *c* > 0 is a constant, sgn⁡(*x*) is the sign function, and Δ*G*_*σ*_ × *I* denotes the image convolved with a Gaussian smoothing filter and calculated by Laplace operator. The associated level set evolution equation is given by

(4) ΔϕADPLS={α[∇2ϕ−div⁡(∇ϕ|∇ϕ|)]+βδε1(ϕ)div⁡[g(∇I)∇ϕ|∇ϕ|]+v(I)g(∇I)δε1(ϕ)}·ΔtADPLS,

where Δ*t* ^ADPLS^ is the time step; the definition of *δ*_*ε*_1__ is given by

(5) $$\begin{array}{c} \delta_{\varepsilon_{1}}\left(x\right)=\frac{1}{\pi }\frac{\varepsilon_{1}}{{\varepsilon_{1}}^{2}+x^{2}}. \end{array}$$

It is worth mentioning that the variable weighting coefficient *v*(*I*) in (4) plays a key role in the evolution of the zero level curves. First, it adaptively guides the zero level curves to the target contour according to the image information. Second, the size of it is adjusted adaptively according to the spatial image information, which can greatly improve the capability of zero level set to detect the edge of multicontour and concavities. Third, by adjusting the size of coefficient *c*, the method can control the zero level curves' ability of capturing the target boundaries. If multicontours exist in the image, we give the coefficient *c* a larger value. On the contrary, if the content of the image is simple, a smaller value is set to coefficient *c*.

Although the ADPLS method has the above advantages, it also has disadvantage as follows. In the segmentation of the images with intensity inhomogeneity, the intensity in one place may vary dramatically from another place, no matter how the edge indicator function *g*(*I*) is adjusted; it also has the possibility that the decrease of *g*(*I*) is too quickly for one place but too slowly for another one. The situation can be seen from Figure 1(b).

### 2.2. The LBF Model

Recently Li et al. [14] proposed the LBF model, which can overcome the segmentation error brought by intensity inhomogeneity using two fitting functions *f*_1_(*x*) and *f*_2_(*x*) which locally approximate the intensities outside and inside the contours. They extracted the object by minimizing the following energy function:

(6) εLBF(C,f1,f2) =λ1∫[∫out(C)kσ(x−y)|I(y)−f1(x)|2dy]dx  +λ2∫[∫in(C)kσ(x−y)|I(y)−f2(x)|2dy]dx  +v|C|+μP,

where *k*_*σ*_ is a Gaussian kernel with standard deviation *σ*. The first two terms in (6) are the weighted mean square error of the approximation of the image intensities *I*(*y*) outside and inside the contour *C* by the fitting values *f*_1_(*x*) and *f*_2_(*x*), respectively, with *K*(*x* − *y*) as the weight assigned to each intensity *I*(*y*) at *y*. The third term |*C*| is the length of the contour, and the last term *P* is the regularization term for level set evolution. *λ*_1_ > 0, *λ*_2_ > 0, *μ* > 0, and *v* > 0 are constants. The associated level set evolution equation is given by

(7) ΔϕLBF={−δε2(ϕ)(λ1e1−λ2e2)+vδε2(ϕ)div⁡(∇ϕ|∇ϕ|)+μ·[∇2ϕ−div⁡(∇ϕ|∇ϕ|)]}·ΔtLBF,

where Δ*t* ^LBF^ is the time step, and the definitions of the edge indicator function *e*_1_, *e*_2_, *f*_1_, and *f*_2_ are given by

(8) ei(x)=∫Ωkσ(y−x)|I(x)−fi(x)|2dy, i=1,2f1(x)=kσ(x)∗[Hε(ϕ(x))I(x)]kσ(x)∗Hε(ϕ(x)),f2(x)=kσ(x)∗[(1−Hε(ϕ(x)))I(x)]kσ(x)∗(1−Hε(ϕ(x))),δε2(x)=1πε2ε22+x2,Hε(x)=12[1+2πarctan(xε)],Hε′(x)=δε(x).

**Figure 2:**
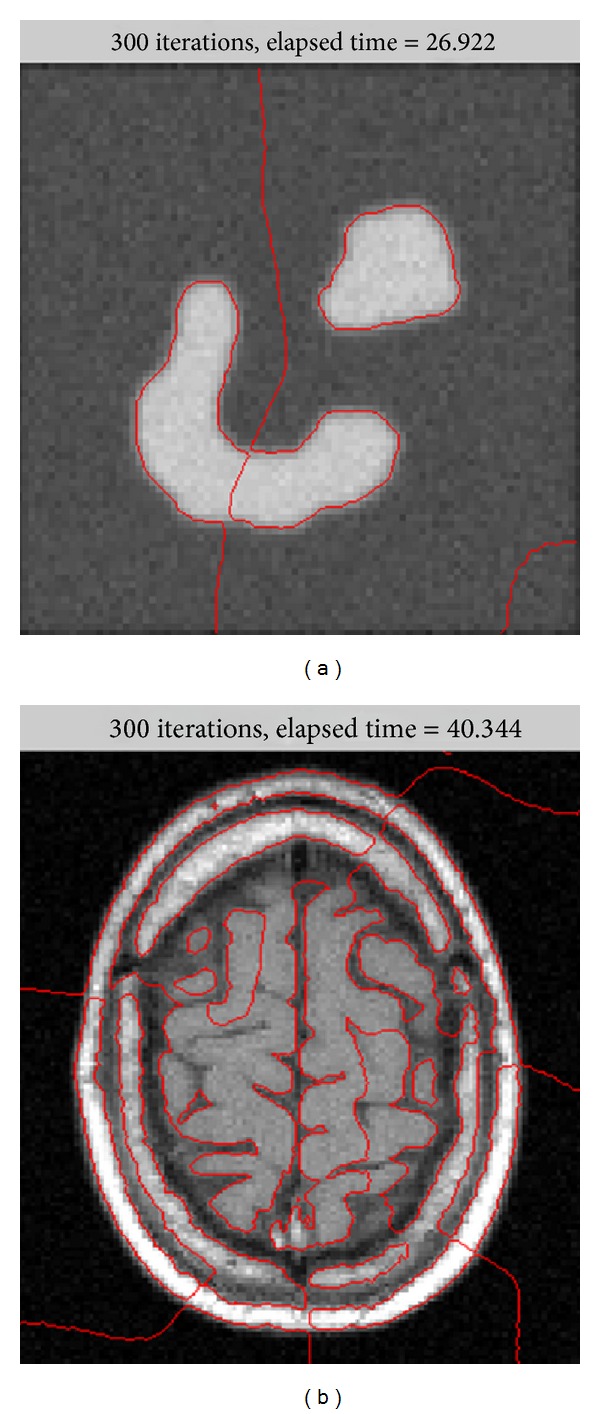
Failure segmentation of LBF from inappropriate initial contour.

Because of the localization property of the kernel function *k*_*σ*_(*x* − *y*), the contribution of the intensity *I*(*y*) to the fitting energy *ε* ^LBF^ decreases to zero as the point *y* goes away from the center point *x*. This localization property plays a key role in segmenting the image with intensity inhomogeneity. And a better result than that of the ADPLS method can be observed from Figure 1(c). However, many local minimums of the energy functional might be simultaneously introduced by this localization property, which means that the LBF model might be sensitive to initial curve, and inappropriate initial curve might lead to the failure of segmentations.  Figure 2 shows the failure situations of the segmentations with inappropriate initial contours. Besides, the limitation about time step limits the speed of the LBF model in the evolution of zero level set.

## 3. The Proposed Method

The proposed method is a fusion method which combines the advantages of the LBF model and the ADPLS method by taking both local and global intensity information into account. It is built to apply the two methods simultaneously in a linear combined form with weights of each method determined by the local image information around each individual pixel.

### 3.1. The Fusion Method

The proposed method updates the level set function through adjusting the variable weighting coefficient between ADPLS method and LBF model as follows:

(9) $$\begin{array}{c} \Delta \phi =w\left(I\right)\cdot \Delta \phi^{\text{ADPLS}}+\left(1-w\left(I\right)\right)\Delta \phi^{\text{LBF}}. \end{array}$$

To make the proposed method adjust adaptively to image information, the value of *w*(*I*) should consider two aspects. Firstly, the variable weighting coefficient should reflect the degree of intensity homogeneity. Secondly, the proposed method should use ADPLS method mainly to be fast and robust when the zero level set evolves in the intensity homogeneity area and use LBF model mainly to segment image accurately when the zero level set evolves near the objective boundary. According to these points above, the variable weighting coefficient *w*(*I*) is designed as follows:

(10) $$\begin{array}{c} w\left(I\right)=\exp\left(-\frac{\sigma \left(I\right)}{d}\right), \end{array}$$

where *d* > 0 is a constant and *σ*(*I*) is the standard deviation of the pixel in a 3 × 3 neighborhood as a matter of experience, which is inversely proportional to the homogeneity of the image in the neighborhood.

**Figure 3:**
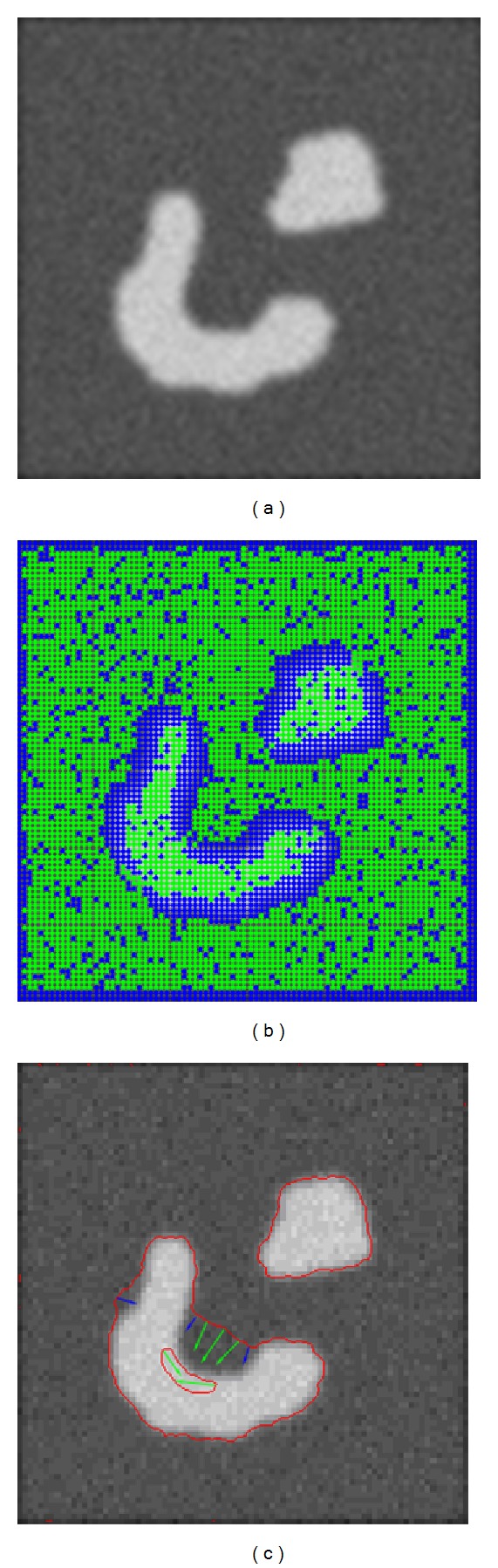
The illustration of zero level set evolution force in the proposed method. (a) The original image. (b) The dominant force of each pixel. (c) The dominant force on the zero level set curve.

The more homogeneous the image's intensity level is, the smaller the value of *σ*(*I*) is. With bigger weight, the ADPLS method will lead the evolution of level set. There are two advantages of the proposed method. First, with the global search ability, the proposed method can avoid being trapped into local minimums. Second, as the ADPLS method, which has a much larger time step, is much faster than the LBF model, the proposed method will be faster than the LBF model. On the other hand, the value of *σ*(*I*) will be bigger when the pixel is near or on the edge. Accordingly, the weight of the LBF model will increase and be even larger than that of the ADPLS method. So the accuracy of the result will be better than the ADPLS method alone. As discussed above, the proposed method combines the advantages of the ADPLS method and the LBF model. This can be seen from a simple experiment of a simulated image shown in Figure 3. Figures 3(b) and 3(c) show the dominant force of the zero level set evolution on each pixel. As can be seen, the dominant force near the real edge that comes from the LBF model is marked blue (the borders of the image with 3-pixel width are set blue). On the other intensity-homogeneous area, the dominant force that comes from the ADPLS method is marked green.

### 3.2. Implementation of Level Set

The implementation steps of the proposed algorithm in this paper can be described as follows.

**Step 1 .**

According to the given arbitrarily region *R* in the image domain, initialize the level set function *ϕ*_0_. If the pixel (*x*, *y*) belongs to the region *R*, then set *ϕ*_0_(*x*, *y*) = −2; otherwise set *ϕ*_0_(*x*, *y*) = 2.

**Step 2 .**

According to the image intensity and (10), we solve the weighting coefficient *w*(*I*) and initialize parameters. Set the variable of iterations iterNum = 0.

**Step 3 .**

If the iterNum is less than the maximum number of iterations iterMax, then repeat the following steps.

**Step 4 .**

Neumann boundary conditions are applied on the boundary of the image. According to the literature [5, 7] and (4) and (7), get Δ*ϕ* ^ADPLS^ and Δ*ϕ* ^LBF^ and then obtain Δ*ϕ* by solving (9).

**Step 5 .**

Update the level set function using the formula *ϕ* = *ϕ* + Δ*ϕ* and set iterNum = iterNum + 1; then return to Step 3.

## 4. Experiments

The proposed method has been tested with both synthetic and medical images from different modalities. Unless otherwise specified, we use the following parameters in this paper. The parameters used in LBF model are Δ*t* ^LBF^ = 0.1, *ε*_2_ = 1.0, *σ* = 3.0, and the parameter of punishment *μ* = 0.002∗255∗255; the values of *λ*_1_ and *λ*_2_ depend on the actual image to be segmented which is described in the literature [14]. The parameters used in ADPLS method are Δ*t* ^GIF^ = 1.0, *ε*_1_ = 1.5, *σ* = 2.0, and the parameter of punishment *β* = 0.2/Δ*t* ^GIF^, *α* = 10; the value of *c* should be small when dealing with simple images and be great with multilayer-complex images, which is referred to in the literature [7]. The constant coefficient *d* in *w*(*I*) of (10) is set by experience. It is appropriate to set *d* between 1 and 10 according to a lot of experiments. When the image is intensity inhomogeneous, *d* should be small and be great on the contrary. We do all the experiments with Matlab code run on a Dell Optiplex 210L PC, with Pentium 4 processor, 3.0 GHZ, 1 GB RAM, with Matlab 6.5 on Windows XP.

**Figure 4:**
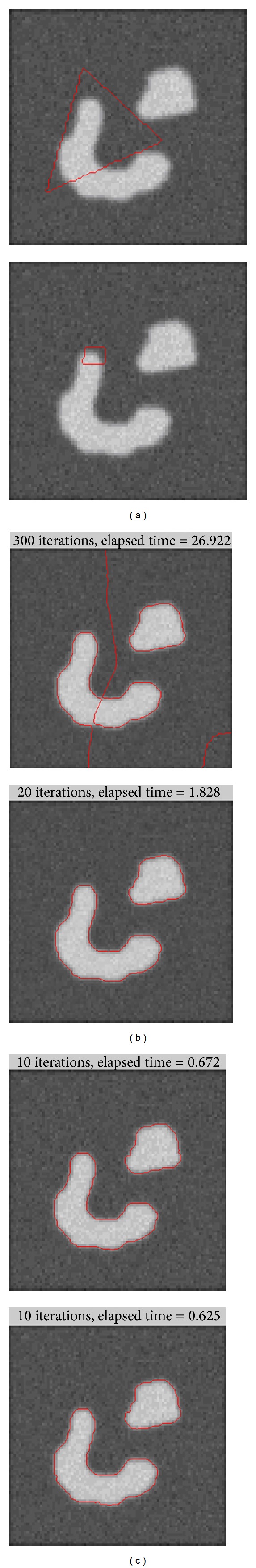
Results of the proposed method and the LBF model for the same synthetic image. Column 1: initial contour and original image. Column 2: final contours of the LBF model. Column 3: final contours of the proposed method.

Figure 4 shows the results of the proposed method and the LBF model using the same synthetic image with the same initial contours in the first row and the second row, respectively. Results in the first row show that the LBF model fails to segment the object correctly after 300 iterations and 26.922 seconds. However, satisfactory segmentations can be obtained by using the proposed method with 10 iterations and 0.672 seconds. In these experiments, we find that, no matter what the initial contours are, the proposed method can lead to better segmentations with less iterations and time costs than the LBF model.

**Figure 5:**
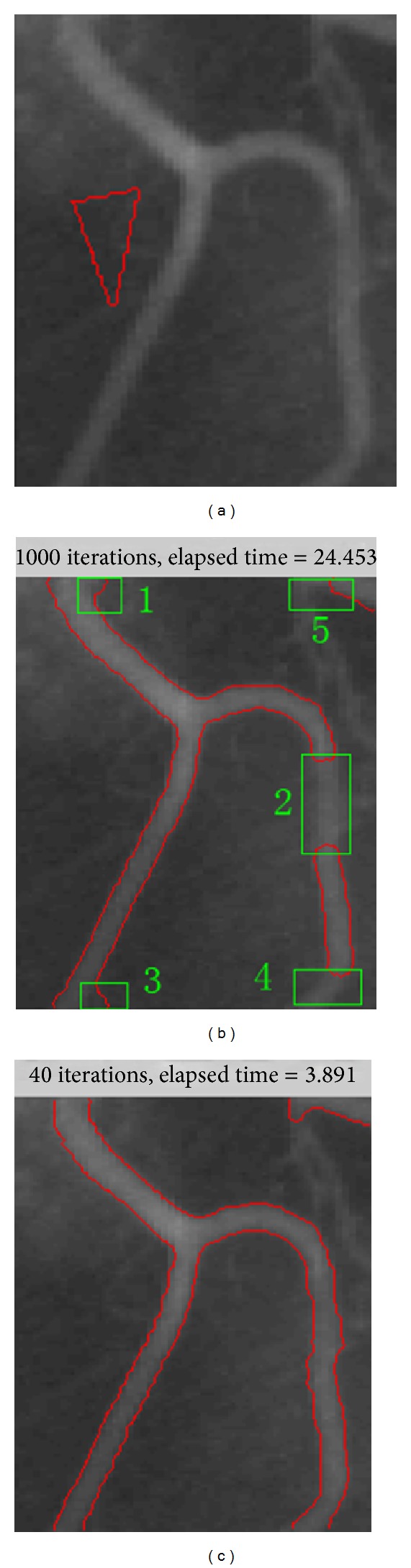
Results of the ADPLS method and the proposed method for a typical image with intensity inhomogeneity. (a) Initial contour and original image. (b) Result of the ADPLS method. (c) Result of the proposed method.

Figure 5 shows the results for an X-ray vessel image, which is a typical image with intensity inhomogeneity. In our experiments, the ADPLS method fails to segment the object correctly after 1000 iterations and 24.453 seconds. As can be seen from Figure 5(b), boundary leakage and lack of segmentation exist simultaneously because of the intensity inhomogeneity. After 40 iterations and 3.891 seconds, the proposed method can achieve satisfactory segmentation results (shown in Figure 5(c)). That weak part of the vessel boundaries can be segmented successfully using the proposed method. This demonstrates that, owing to combining LBF model, the proposed method is more accurate than the ADPLS method.

**Figure 6:**
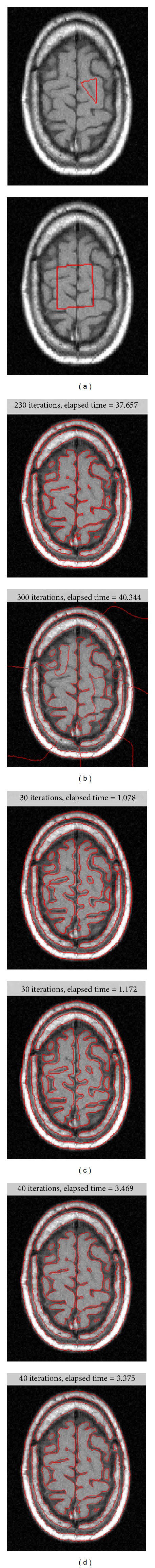
Results of the three methods for a MR image. (a) Initial contour and original image. (b) Result of the LBF model. (c) Result of the ADPLS method. (d) Result of the proposed method.

**Figure 7:**
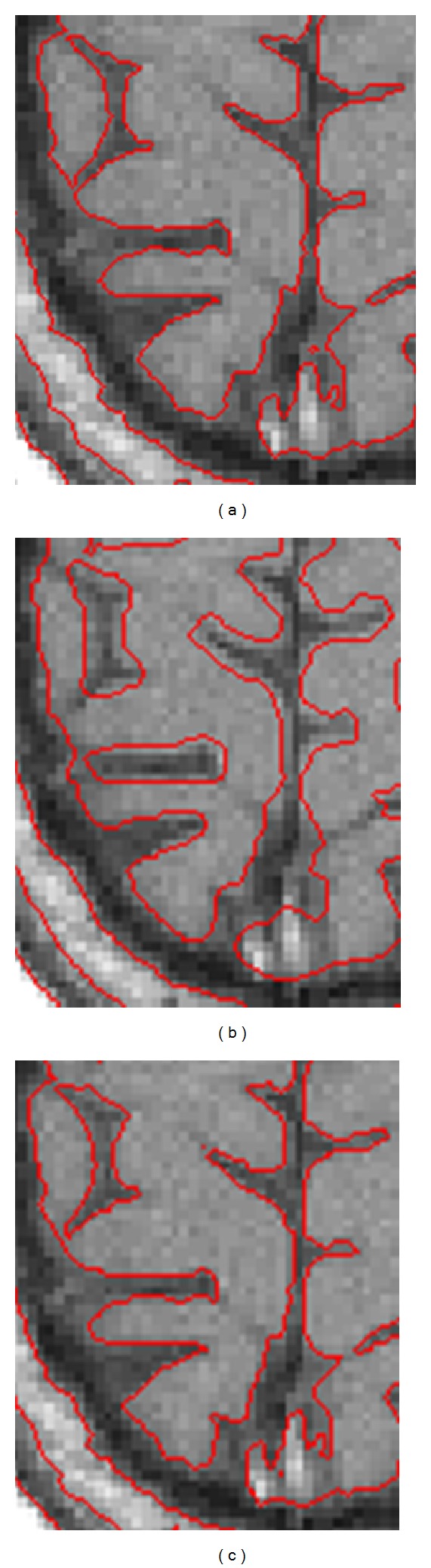
Enlarged views of segmentation results on local region for row 1 in Figure 6. (a) Result of the LBF model. (b) Result of the ADPLS method. (c) Result of the proposed method.

Figure 6 shows the segmentation results for a brain MR image using the three methods mentioned above. Column 1 shows the initial contours, and Columns 2, 3, and 4 show the results of the LBF model, the ADPLS method, and the proposed method, respectively; they all have the same initial contours in column 1.  Figure 7 gives the enlarged view of segmentation results on local region of row 1 in Figure 6. As to the segmentations in Figures 6 and 7, the stability, iterations, elapsed time, and accuracy are listed in Table 1. Regarding accuracy, the proposed method is close to the LBF model and much more accurate than the ADPLS method. With regard to elapsed time, the proposed method is close to the ADPLS method but much faster than the LBF model. With regard to algorithm stability, the proposed method and the ADPLS method are less sensitive to the initialization than the LBF model.

**Figure 8:**
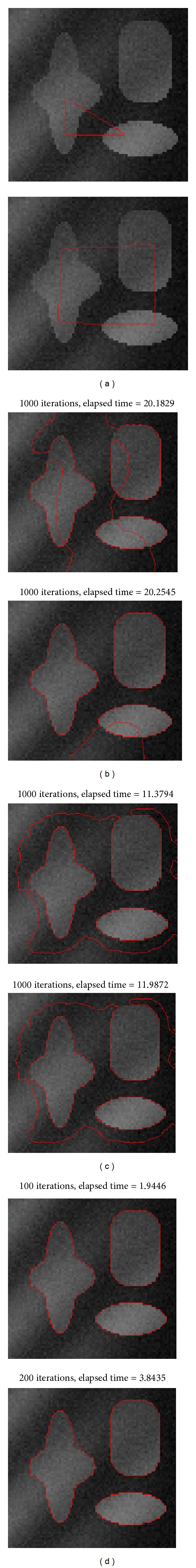
Results of the three methods for a synthetic image. (a) Initial contour and original image. (b) Result of the LBF model. (c) Result of the ADPLS method. (d) Result of the proposed method.

Figure 8 shows the segmentation results for a synthetic image using the three methods mentioned above. Column 1 shows the initial contours, and Columns 2, 3, and 4 show the results of the LBF model, the ADPLS method, and the proposed method, respectively; they all have the same initial contours in Column 1. The extensive experimental results showed the superior performance of the proposed method over the state-of-the-art methods, in terms of both robustness and efficiency.

## 5. Conclusions

The fusion method proposed in the paper uses a variable weighting coefficient to combine both ADPLS method and LBF model. The proposed method allows the ADPLS method to be dominant force in intensity-homogeneous area of the image and the LBF model to be dominant force in intensity inhomogeneous area and objective boundaries, so it could take full use of these two algorithms' advantages, respectively, to complement each other. Compared with the LBF model, the fusion method is less susceptible to the initial contours and can always get good segmentation results and can be much faster. On the other hand, compared with the ADPLS, the proposed method can fully avoid boundary leakage and lack of segmentation with high speed when processing images with intensity inhomogeneity. Experiments show that the present method has superior comprehensive performance compared to ADPLS and LBF algorithm, respectively, in segmentation accuracy, speed, and stability.

## Figures and Tables

**Table 1:** The performance comparison of three methods for complex image segmentation.

	Stability	Iteration	Elapsed time	Accuracy
The LBF model	No	230 times	37.657 seconds	High
The ADPLS method	Yes	30 times	<1.20 seconds	Low
The proposed method	Yes	40 times	<3.5 seconds	High
